# Influenza Vaccination in Identified People Living with HIV/AIDS and Health Care Providers of Triangular Clinics in Iran, 2015–2016

**Published:** 2018-02

**Authors:** Mohammad Mehdi GOUYA, Katayoun TAYERI, Hossein AKBARI, Farshid REZAEI, Peyman HEMMATI, Mahmood SOROUSH

**Affiliations:** 1. Center for Communicable Disease Control, Ministry of Health & Medical Education, Tehran, Iran; 2. Iranian Research Center of HIV & AIDS, Tehran University of Medical Sciences, Tehran, Iran; 3. Surveillance Department, Center for Communicable Disease Control, Ministry of Health & Medical Education, Tehran, Iran

## Dear Editor-in-Chief

Influenza is one the main causes of respiratory illness in people living with HIV/AIDS (PLWH), but there is insufficient knowledge about the clinical course, severity, and consequences of pandemic H1N1 2009 in these patients. H1N1 2009 circulated during 2015–2016 throughout Iran.

Influenza usually has a benign and self-limited course in general population but sometimes leads to moderate to severe complications. Sinusitis, bronchitis, and pneumonia are some of the known complications of Influenza and may need to hospitalization (1). Influenza is one of the main causes of exacerbating the underlying diseases like asthma and congestive heart failure. At risk groups for influenza are children <2, old age >65 yr, pregnant women, residents of nursing home, individuals with neurologic disorders, chronic pulmonary and cardiovascular diseases and PLWH (1, 2).

Influenza vaccination is recommended as a preventive strategy for at-risk people. Several studies showed destructive effects of influenza on PLWH and acceptable flu vaccine efficacy on them (3). Incidence of influenza in vaccinated and unvaccinated PLWH during flu season was 6% and 21% respectively (4). In a cohort study, 3 patients from 26 PLWH who infected with influenza were admitted in ICU (5). ICU admission rate in PLWH with influenza was 10% and 4.7% in another study (5–7). In two studies hospitalization rate of PLWH infected with influenza were about 33% and 54% respectively (7, 8).

In Iran, counseling center for behavioral diseases (triangular clinics, TC) are the main centers which provide regular and free of charge HIV/AIDS care and treatment services. Trivalent flu vaccination is provided annually to all PLWH and TCs' staff. In this study, we investigated flu vaccination coverage in PLWH and its complication, severe acute respiratory illness (SARI), hospitalization and death rate in PLWH and TCs' personals from 20 Nov 2015–22 Jan 2016.

Data were gathered from 49 clinics throughout the country with a questionnaire. Overall, 5336 from 9554 registered PLWH vaccinated with trivalent flu vaccine (56%). Most of un-vaccinated individuals were patients who did not refer to triangular clinics. From 20 Nov to 22 Jan, 177 PLWH reported mild respiratory illness, and 12 patients were hospitalized due to SARI (125/100000). No one need to ICU and no death was reported. Nine from 12 hospitalized patients had history of flu vaccination. Mean and median time of hospitalization was 7 and 9.25 days respectively. From 555 health care providers who were working in TCs, 409 were vaccinated (74%) and one of them hospitalized due to SARI. No one need to ICU and no death was reported.

At the same time, the incidence of SARI in general population was 34.9/100000. Overall, 28576 individuals were hospitalized due to SARI throughout the country which 20726 of them were sampled for influenza virus, 3216 positive result was reported. Totally, 1047 patients died from severe respiratory illness which 254 of them had positive results for influenza.

Influenza vaccine complication is very low. Some of the mild complication are redness/pain of injection site, low-grade fever, light headache, nausea and myalgia (9). Very low possibility of relation between Guillain-Barre syndrome (GBS) and influenza vaccination have been considered but this relation was not approved by many other studies (10). Indeed, incidence of GBS after influenza is much more likely than developing GBS after influenza vaccination (9). In this study, no major complication from influenza vaccination was reported in PLWH and health care providers. Health care providers of TCs should be vaccinated annually. Interview with un-vaccinated personals showed that there is some misconception about influenza vaccine and its complications, including GBS, which is the leading cause of unwilling to flu vaccination.

For reducing the misconception about influenza vaccine, especially among healthcare providers, it is necessary to hold training course for them and update their information about the efficacy and importance of influenza vaccination. Obviously, trained personals can have more willing to encourage the PLWH to be vaccinated.

Although despite limitations, we can conclude that vaccination of PLWH can protect this high-risk group against influenza infection and its severe consequences.
